# An Atypical Presentation of Primary Hyperparathyroidism in an Adolescent: A Case Report of Hypercalcaemia and Neuropsychiatric Symptoms Due to a Mediastinal Parathyroid Adenoma

**DOI:** 10.3389/fendo.2020.581765

**Published:** 2020-09-29

**Authors:** Roberta Minelli, Aniello Meoli, Alessandra Tiri, Umberto Fanelli, Rosanna Iannarella, Pierpacifico Gismondi, Susanna Esposito

**Affiliations:** ^1^Thyroid Unit, Medical Clinic, Department of Medicine and Surgery, University of Parma, Parma, Italy; ^2^Pediatric Clinic, Pietro Barilla Children's Hospital, Department of Medicine and Surgery, University of Parma, Parma, Italy

**Keywords:** hypercalcaemia, mediastinal parathyroid adenoma, neuropsychiatric symptoms, primary hyperparathyroidism, psychiatric disorders

## Abstract

Psychiatric disorders are rare clinical manifestations of hypercalcaemia in the pediatric population, are relatively more frequent during adolescence and are often overlooked in cases of severe hypercalcaemia. We described the case of a 17-year-old girl affected by anorexia nervosa, depression and self-harm with incidental detection of moderate hypercalcaemia. Clinical, laboratory and instrumental tests demonstrated that hypercalcaemia was secondary to primary hyperparathyroidism (PHPT) due to a mediastinal parathyroid adenoma in the thymic parenchyma. After parathyroidectomy with robot-assisted surgery, we observed the restoration of calcium and PTH levels in addition to an improvement in psychiatric symptoms. This case demonstrates that serum calcium concentration should be evaluated in adolescents with neurobehavioural symptoms and in cases of hypercalcaemia PHPT should be excluded. Surgery represents the cornerstone of the management of PHPT and may contribute to improving quality of life and psychological function in these patients. However, the complexity of neurological involvement in cases of hypercalcaemia due to PHPT requires further investigations to establish the real impact of this condition on the neurocognitive sphere.

## Background

Hypercalcaemia, a serum calcium concentration greater than two standard deviations above the normal mean for sex and age, is rare in childhood. It is classified, according to total serum calcium concentrations, as mild (<12 mg/dL), moderate (between 12 and 14 mg/dL), and severe (>14 mg/dL) (1). The causes of hypercalcaemia are different and age-specific, and many of them develop within the hereditary syndromes caused by germline mutations (2). The different etiologies of hypercalcaemia can be divided into two categories: PTH-dependent and PTH-independent (e.g., etiologies related to vitamin D, malignancy, medications, other endocrine disorders such as hyperthyroidism and pheochromocytoma, and genetic disorders such as familial hypocalciuric hypercalcaemia and other causes such as prolonged immobilization) (3).

The evaluation of a patient with hypercalcaemia includes a medical history, a physical examination and laboratory tests, including the measurement of total calcium, ionized calcium, PTH, 25-hydroxyvitamin D and serum creatinine levels in addition to the determination of 24-h urine calcium and creatinine levels. When hypercalcaemia is associated with elevated PTH, the most likely diagnosis is primary hyperparathyroidism (PHPT) (4). Primary hyperparathyroidism is usually sporadic during childhood or adolescence, and in 65% of cases, it is due to a single parathyroid adenoma. Moreover, cases of neurocognitive complications in children and adolescents have not been previously described. Although the majority of these adenomas are located in the cervical region, 11–25% of them are located near the mediastinum and 1–2% are located within the mediastinum. In the age group between 3 and 19 years, the average age of onset was 12.8 years, with a female:male ratio of 3:2. Patients with multi-gland disease usually have genetic syndromes (MEN1 and MEN2a) (2, 5). The biochemical features of PHPT are hypercalcaemia, hypercalciuria, hypophosphatemia and inappropriately normal or elevated PTH concentrations (6).

Hypercalcaemia may be associated with different clinical manifestations; depending on its severity, it can be totally asymptomatic or it can show renal, gastrointestinal, musculoskeletal and neuropsychiatric symptoms. A spectrum of neuropsychiatric disorders (e.g., anxiety, depression, cognitive dysfunction) have been associated with hypercalcaemia, mostly in patients with PHPT. On the basis of neuropsychological complaints induced by hypercalcaemia, the mechanisms of PHPT are unclear, but they may be determined by glutamate neurotoxicity via NMDA receptor activation or by changes in monoamine levels in the central nervous system (7, 8). Improvement in some of these disorders has been described after the correction of HPT. We described the case of a 17-year-old girl affected by anorexia nervosa, depression and self-harm with incidental detection of moderate hypercalcaemia secondary to PHPT due to a mediastinal parathyroid adenoma in the thymic parenchyma.

## Case Presentation

A 17-year-old girl was admitted to the Psychiatric Clinic of Parma because of a refusal to eat manifested in the previous days and a report of suicidal ideations that emerged following stressful environmental and relational events. The patient was followed up by the local child neuropsychiatric service for 1 year for adaptation disorder with mixed anxiety and depressed mood. The patient had a positive history of self-injurious gestures for suicidal purposes and was in treatment with venlafaxine (75 mg/day), lamotrigine (25 mg/day), gabapentin (200 mg/day), and lorazepam (2.5 mg/day) with a poor response to therapy and a family history of depression. No history of neck irradiation as well as treatment with lithium preparations was reported. After 9 days of hospitalization, necessary to reach sufficient a psychopathological state within the limits of the basic personological framework, a transfer to the Pediatric Clinic of our Children's Hospital was requested for the incidental detection of hypercalcaemia. At admission, the subscore at the Hospital Anxiety and Depression Scale was 10.

Upon arrival, the initial laboratory work-up showed elevated calcium level (13.3 mg/dL; normal range: 8.8–10 mg/dL), increased ionized calcium concentration (6.7 mg/dL; normal range: 4.7–5.2 mg/dL) and reduced phosphorus level (2.6 mg/dL; normal range: 2.7–4.5 mg/dL), which is why intravenous fluids and serial ECG recordings were started. The parathyroid hormone levels was 193 pg/mL (normal range: 14–72 pg/ml), while the vitamin D levels was slightly reduced at 21 ng/mL (normal range: 20–79 pg/mL); no other abnormalities were detected in blood tests. Regarding urinary tests performed on 24-h urine collection, they pointed out hypercalciuria at 10.5 mg/kg/day (normal value <4 mg/kg/day), which strongly suggested PHPT excluding familial hypocalciuric hypercalcaemia.

**Figure 1:**
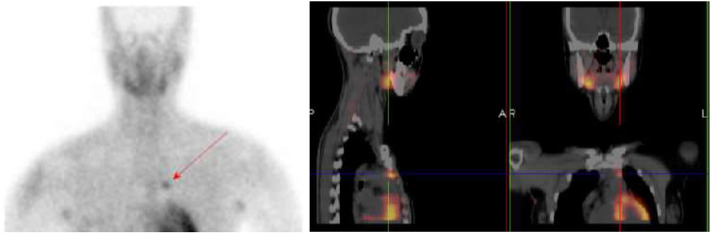
Sesta-MIBI parathyroid scan combined with SPECT-CT study of the neck-mediastinum showing the anterior mediastinal parathyroid adenoma.

The imaging work-up first involved thyroid/parathyroid ultrasonography, which revealed a thyroid of normal size with homogeneous echotexture and no lesions, while the parathyroids were not visible. The subsequent Sesta-MIBI parathyroid scan combined with SPECT-CT fusion study of the neck-mediastinum highlighted an anterior mediastinal parathyroid adenoma localized behind the manubrium body of the sternum (Figure 1).

**Figure 2:**
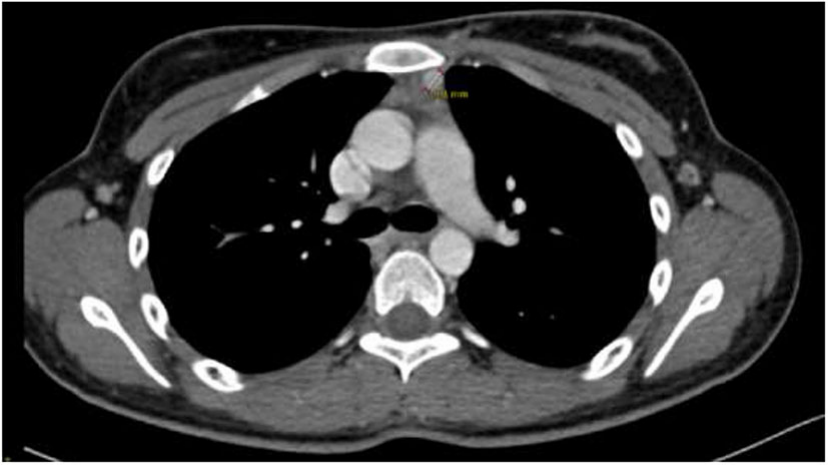
CT scan of the chest showing solid nodularity approximately 9 mm in diameter in the anterior mediastinum.

Therefore, further laboratory and instrumental tests were necessary to exclude the presence of lesions or associated alterations attributable to a MEN framework. For this purpose, neck-thorax-abdomen contrast-enhanced CT was performed, which confirmed the presence of a solid nodularity with a diameter of ~9 mm in the anterior mediastinal site and excluded the presence of further alterations of the studied organs (Figure 2). Brain-pituitary gadolinium-enhanced MRI was also performed and showed a globular aspect in the adenohypophysis with normal pituitary functionality.

The results of blood and urinary tests executed to study calcium-phosphorus metabolism and the pituitary-adrenal, pituitary-thyroid, and pituitary-gonadal axes and to exclude the presence of neuroendocrine tumors (neuron specific enolase, chromogranin A, urinary metanephrines, vanillylmandelic acid, serotonin) or other PHPT possible complications were negative. In order to exclude a multiple endocrine neoplasia, mutation analysis of the *MEN1* and *RET* genes was performed and resulted negative.

During hospitalization, the calcium level remained stable at 11 mg/dL initially with intravenous fluid therapy alone, after which 25 mg/dL furosemide was administered per os. In consideration of the new verbalization of suicidal ideations by the patient, two psychiatric consultations were performed with subsequent re-modulation of the pharmacological treatment (lamotrigine 50 mg/day, gabapentin 100 mg/day), which led to a slight improvement in the psychic framework. Mediastinal parathyroidectomy associated with complete thymectomy was finally performed with a robot-assisted approach (duration of intervention: 3 h) with no perioperative complications and in a few hours led to the normalization of PTH and calcium levels. Histological examination showed a parathyroid adenoma with an organoid pattern composed of medium-sized oval to polygonal cells, a moderate amount of eosinophilic cytoplasm, round to oval centrally placed nuclei, and, in many cells, prominent nucleoli. Although intraoperative PTH was not evaluated, subsequent blood laboratory tests did not highlight further alterations (Table 1). The patient was discharged after 3 days from the intervention. Regarding the neuropsychiatric sphere, during the following investigations, no other suicidal ideations were registered, while a modest improvement in mood, energy and health perception with a subscore of 5 at the Hospital Anxiety and Depression Scale was reported at the 3-month follow-up. Lamotrigine and gabapentin were stopped and no further need of neuropharmacologic treatment was requested at the 6-month follow-up.

**Table 1:** Preoperative and postoperative laboratory work-up results.

**Blood**	**Preoperative**	**Postoperative**	**Reference range**
Red blood count (RBC)	4.52 × 10^6^/μL	3.7 × 10^6^/μL	3.9–5.2
Hemoglobin (Hb)	12.7 g/dL	10.4 g/dL	12–16
Haematocrit (Ht)	38.7%	33.2%	36–46
Mean corpuscular volume (MCV)	85.6 fL	89.7 fL	80–95
Mean corpuscular hemoglobin (MCH)	28.1 pg	28.1 pg	26–34
Platelets (PLT)	226 × 10^3^/μL	205 × 10^3^/μL	150–400
White blood count (WBC)	6.1 × 10^3^/μL	7.69 × 10^3^/μL	4–10
Neutrophils	54.2%	66.6%	40–70
Lymphocyte	39.7%	25.2%	20–40
Blood urea nitrogen	11 mg/dL	24 mg/dL	10–50
Creatinine	0.6 mg/dL	0.6 mg/dL	0.5–1.4
Lactate dehydrogenase (LDH)	92 U/L	–	<248
Sodium	141 mEq/L	140 mEq/L	135–148
Potassium	3.7 mEq/L	3.3 mEq/L	3.5–5.3
Chloride	110 mEq/L	104 mEq/L	96–112
Magnesium	1.9 mg/dL	–	1.6–2.5
Calcium	13.3 mg/dL	8.5 mg/dL	8.3–10.5
Ionized calcium	6.7 mg/dL	4.8 mg/dL	4.7–5.2
Phosphorous	2.6 mg/dL	–	2.5–4.8
Albumin	3.5 g/dL	–	3.5–5
PTH	193 pg/mL	38 pg/mL	14–72
Vitamin D 25	21 ng/mL	–	25–80
**24-h urine collection**			
Calcium	10.5 mg/kg/day	–	<4
Phosphorous	0.5 g/24 h	–	0.7–1.5
Magnesium	61 mg/24 h	–	70–240
Creatinine	1.49 g/24 h	–	1–2.5

## Discussion

PHPT represents one of the main causes of hypercalcaemia and is more common in females. The constellation of symptoms included in cases of hypercalcaemia, in addition to the classic gastrointestinal disorders, bone loss and renal stones, includes non-specific symptoms such as fatigue, muscle weakness, and bone and joint pain; not even the neuropsychiatric sphere is spared since patients with this condition may present psychological and psychiatric symptoms such as insomnia, irritability, a loss of initiative, anxiety, depression, personality changes, cognitive dysfunction (mostly memory), confusion, psychosis and even coma (9, 10). Neuropsychiatric involvement in the case of PHPT has been described since the 1940s and is present in up to 23% of patients, usually after a long period of subclinical hypercalcaemia (9–11). The severity of neuropsychological complaints, which can also appear in cases of mild hypercalcaemia and usually after a period of subclinical hypercalcaemia, do not correlate strictly with its grade, although the risk of acute psychosis increases with increasing concentrations of serum calcium (12). The prevalence of the aforementioned disturbances in samples of PHPT patients undergoing parathyroidectomy was ~51.9% for anger and irritability, 33–62.1% for depression, 43.1–53% for anxiety, 22% for thoughts of death or suicide, 5–20% for hallucinations and delusions and 37.3–46.5% for cognitive impairment (9, 13). However, neurocognitive complications as distinctive features of PHPT have not been previously reported in children and adolescents. Surgical management of mild PHPT at the time of diagnosis has been associated with improved neuropsychological symptoms (14).

We presented the case of a 17-year-old girl hospitalized for a refusal to eat in association with the manifestation of suicidal ideations; the patient had been in neuropsychiatric follow-up for 1 year because of adaptation disorder with mixed anxiety and depressed mood and a history of suicide attempts. In the patient's medical history, different treatments were reported with poor response to psychotherapy and psychotropic drugs. Upon admission to the ward, she was in therapy with venlafaxine, lamotrigine, gabapentin and lorazepam. Laboratory and instrumental work-up allowed us to make the diagnosis of PHPT successively attributable to a solitary parathyroid adenoma located in thymic parenchyma, an uncommon location for this type of lesion (1–2% of cases), that was removed with robot-assisted surgery. A consequent restoration of serum PTH and calcium levels was demonstrated in postoperative laboratory blood tests.

The paradigm that only the presence of typical symptoms related to PHPT is an indication for surgery has been refuted by the latest studies. In the case of atypical presentation of PHPT with only neuropsychiatric symptoms and even in the case of mild hypercalcaemia, the importance of surgery as the only definitive therapy for this condition is outlined by the most recent scientific. In a prospective study, the authors evaluated depressive symptoms using the Patient Health Questionnaire-9 (PHQ-9) and anxiety symptoms using the Generalized Anxiety Disorder-7 (GAD-7), achieving improved scores after parathyroidectomy (15). Another prospective study obtained an improvement in terms of fatigue, anxiety, depression and sleep-related impairment in PHPT patients after parathyroidectomy as measured by the Patient-Reported Outcomes Measurement Information System (PROMIS) (16). Some previous studies had already described the effect of surgery in these patients, which was associated with a decline in anxiety and depression as well as a significant improvement in health-related quality of life (HRQOL) in the first year. In addition, parathyroidectomy was also associated with a 51% reduced prevalence of suicidal ideation (17, 18). Unfortunately, we did not perform intraoperative PTH. Although robot-assisted surgery appeared successful in our case, a recent study showed that the use of intraoperative PTH monitoring is useful to reduce the risk of unsuccessful surgery (19).

## Conclusion

This case demonstrates that serum calcium concentration should be evaluated in adolescents with neurobehavioural symptoms and in cases with hypercalcaemia PHPT should be excluded. Surgery represents the cornerstone of the management of PHPT and may contribute to improving quality of life and psychological function in these patients. However, the complexity of neurological involvement in cases of hypercalcaemia due to PHPT requires further investigations to establish the real impact of this condition on the neurocognitive sphere.

## Data Availability Statement

All datasets presented in this study are included in the article/supplementary material.

## Ethics Statement

The Ethics Committee of Area Vasta Emilia Romagna Nord approved the publication of this case report and both parents and the participant provided written informed consent for the publication of this manuscript, including photography.

## Author Contributions

RM performed the diagnosis and was in charge of the patient's follow-up. AM and AT wrote the first draft of the manuscript and participated in the patient's management. UF and RI performed the literature review. PG was in charge of the patient's management at admission. SE supervised the patient's management, provided scientific contributions, and critically revised the paper. All authors contributed to the article and approved the submitted version.

## Conflict of Interest

The authors declare that the research was conducted in the absence of any commercial or financial relationships that could be construed as a potential conflict of interest.
